# Learning styles of physiotherapists: a systematic scoping review

**DOI:** 10.1186/s12909-018-1434-5

**Published:** 2019-01-03

**Authors:** Jessica Stander, Karen Grimmer, Yolandi Brink

**Affiliations:** Division of Physiotherapy, Faculty of Medicine and Health Sciences, Stellenbosch University, Francie van Zijl Drive, Tygerberg, Cape Town, 7505 South Africa

**Keywords:** Learning style, Physiotherapy, Allied health

## Abstract

**Background:**

Understanding students’ learning styles, and modifying teaching styles and material accordingly, is an essential to delivering quality education. Knowing more about the learning styles of physiotherapy learners will assist educators’ planning and delivering of learning activities. The purpose of this scoping review was to explore what is published about physiotherapy learning styles.

**Methods:**

An adapted Arksey and O’Malley framework was applied to undertake this systematic scoping review. Nine electronic databases (CINAHL, BIOMED CENTRAL, Cochrane, Web of Science, PROQUEST, PubMed, OTseeker, Scopus, ERIC) were searched using the keywords: ‘learning styles’ and ‘physiotherapy’. English-language, primary research articles that investigated physiotherapy learners’ learning styles were sought.

**Results:**

Of 396 potentially-relevant articles, 15 were included in this review. The studies mostly reflected undergraduate students (910 undergraduates, 361 postgraduates, 23 professionals), in developed countries. Nine articles used the Kolb’s experiential learning theory (ELT); one study applied Honey and Mumford’s approach; two studies used the Gregorc model of cognition and three studies did not specify an underlying theory. Outcome measures included different versions of Kolb’s Learning Style Inventory, the visual-aural-read/write-kinesthetic questionnaire, Gregorc style delineator, Felder Silverman’s Index of Learning Survey, and Honey and Mumford’s Learning Style Questionnaire.

The preferred physiotherapy learning styles, according to the ELT, seem to be Converger (learns “hands-on” and applying previously attained knowledge) and Assimilator (gathers and organises information to make the most sense).

**Conclusions:**

Both physiotherapy learners and physiotherapists have specific learning styles of active participation, underpinned with practical examples of theoretical concepts. More research is needed in developing countries, and on postgraduate and professional physiotherapy learners’ learning styles. Also, further research should focus on defining and describing physiotherapy learning styles in a way to be used as an industry standard; and developing valid and reliable learning style outcome measures applicable across physiotherapy learners and settings.

**Electronic supplementary material:**

The online version of this article (10.1186/s12909-018-1434-5) contains supplementary material, which is available to authorized users.

## Background

There is an increasing international drive to educate healthcare professionals to implement research evidence into practice, known as knowledge translation [1]. To stay abreast of current information, continuous learning is essential for anyone working in the health industry [2]. Providing knowledge translation training tailored to different learning styles and needs, ensures cost effective and efficient use of time, resources and learning opportunities to improve discipline-specific uptake of evidence-based practice [3].

Learning styles and learning outcomes of health science students have been researched since the 1970s [4]. Health science students consist of undergraduate and postgraduate students, and professionals undertaking continuing professional development courses. As most health science students are aged over 18 years, research has focused on adult learning. This is also the age of legal recognition of adulthood in most countries [5]. For this review, the term “learning styles” is defined as “characteristic cognitive, affective, and psychosocial behaviours that serve as relatively stable indicators of how learners perceive, interact with, and respond to the learning environment” (page 4) [6].

It has been postulated that educators who recognise, understand and respond to the learning styles of their students, assist optimal learning and retention of important concepts and information [7]. However, there is no clear correlation between learning styles and subsequent knowledge acquisition [8]. Also, a preferred learning style does not imply the only way in which that individual learns [8].

Staying up-to-date with current knowledge about PT learning styles may assist PT educators to better plan and deliver learning activities. By incorporating discipline-specific learning activities and instructing methods into teaching programs, such learning opportunities may assist PT learners to expand their learning opportunities, and optimise their learning experiences and outcomes [8]. Adult learner outcomes refer to “statements that describe significant and essential learning that learners have achieved, and can reliably demonstrate at the end of a course or program” [9]. Exploring learning outcomes is not within the scope of this review but helps to frame the importance of learning styles to assist in reaching the desired learning outcomes.

**Fig. 1:**
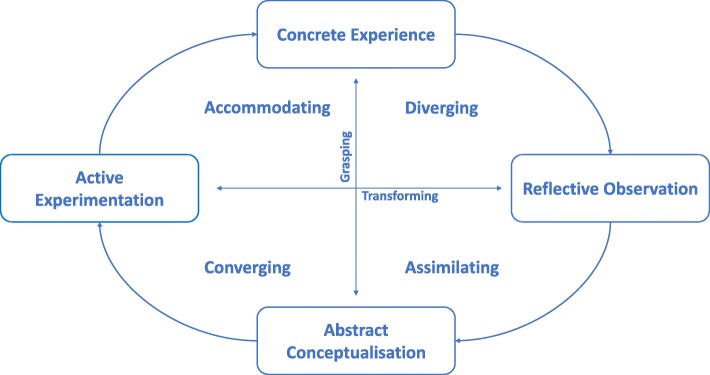
Kolb’s Experiential learning theory cycle (adapted with permission from Kolb & Kolb 2012)

Adult learning theories can be categorised as instrumental learning theories, humanistic theories, transformative learning theories, social theories of learning, motivational models, and reflective models [10]. This review briefly introduces the educator to experiential learning, categorised as an instrumental learning theory [10]. Experiential learning refers to the “learning by doing” approach whereby the learner actively engages cognitively, affectively and behaviourally to assimilate and apply the presented learning material to create new knowledge [11–14]. It focusses on early integration of clinical learning opportunities in physiotherapy curriculum [12–14]. Kolb’s experiential learning theory (ELT) is one of the most widely accepted learning theories [15]. The ELT model is presented in a cycle (Fig. 1), focussing both on the grasping and transforming experience of learning. This is presented through a Y (concrete experience (CE; feeling) versus abstract conceptualisation (AC; thinking)) and X axis (active experimentation (AE; doing) versus reflective observation (RO; watching)) respectively. It is surrounded by a cycle, presenting learning taking place through all four quadrants (Diverger, Assimilator, Converger and Accommodator) and the continuation of that learning process by experiencing, reflecting, thinking and acting [15, 16].

- The Diverger (CE and RO) experiences and then reflects on a situation from different perspectives at a later stage.
- The Assimilator (AC and RO) gathers and organises information to make most sense.
- The Converger (AC and AE) learns “hands-on” and applying previously attained knowledge.
- The Accommodator (CE and AE) is also “hands-on”, but wants to find the solution through trial-and-error [16].

The Learning Style Inventory (LSI) is used to evaluate the individual’s learning style according to the ELT [17]. The LSI’s validity and reliability has been found to be inconsistent, but it has been used in diverse disciplines, ranging from health sciences and engineering to arts and economics [16, 18].

The Honey and Mumford theory, based on Kolb’s ELT with their classifications corresponding to those defined by Kolb [19]. Their Learning Style Questionnaire (LSQ) have been used mostly in business environments and classifies individuals into:

- activists (experiential learners),
- reflectors (reflecting on observations),
- theorists (learning through theoretical concepts)
- pragmatics (actively participating and learning by doing) [8].

Felder and Silverman developed the Index of Learning Survey (ILS), initially for use in engineering education, but it has also been found to be valid among medical students [20, 21]. The ILS classifies individuals into four areas: preference to information observation (sensory or intuitive; visual or verbal), active versus reflective processing of information, and sequential versus global progression to understanding information [8]. The Gregorc model of cognition reflects Gregorc’s view of learners’ inherent inclination to a specific learning style, but that each learner also needs to be able to function within the other learning styles if required by the teaching material [19]. The Gregorc style delineator categorises learners as: Concrete/Sequential (ordered sequence in learning with concrete examples), Abstract/Sequential (verbal and analytical), Abstract/Random (group activities and reflective), Concrete/Random (experimentation and problem-solving) or dual (combination of learning styles) [22].

Culture influences learning styles through a change in the way information is processed and utilised [23]. However, culture does not reside within countries alone, and may be more relevant towards different professional disciplines [24]. There are a range of disciplines included under the allied health (AH) umbrella, including allied (therapies) and scientific professions [25]. The therapies generally consist of physiotherapy (PT), occupational therapy, speech pathology, clinical nutrition and podiatry [25]. AH therapies have been found to have different preferred learning styles, which may influence the most effective teaching and learning approaches for students in these disciplines [26]. On this basis, it is unlikely that there is a ‘one size fits all’ AH learning style. Milanese et al. (2014) found that AH professionals learn better through a blended learning approach, particularly if program design addresses variability in student learning styles and educational needs [27]. Blended learning is a combination of different teaching techniques, technology and learning approaches, which may assist AH learners to bridge the theory-to-clinical implementation gap to improve practice behaviours [28, 29].

This review focuses on adult PT learners (undergraduate and postgraduate students, physiotherapists seeking professional development opportunities). The aims of this systematic scoping review were to:

- Identify how learning styles of PT adult learners were described in the literature;
- Describe the ways in which learning style outcome measures were captured;
- Explore different teaching and learning approaches on learning styles;
- Explore the demographics of the studies; and
- Identify gaps in the current literature relating to PT learning styles.

## Methods

### Study design

A systematic scoping review was conducted. This consists of a systematic search, literature evaluation and descriptive synthesis of current research evidence for a broad topic, using qualitative and quantitative methods [30].

### Quality framework and reporting standard

An adapted standard scoping review framework was followed to ensure a systematic search, literature evaluation and descriptive synthesis of current research evidence [30, 31]. This review included: 1) identifying research questions; 2) identifying relevant studies; 3) study selection; 4) charting the data; and 5) collating, summarising and reporting the results. Reporting on this scoping review followed the PRISMA Extension for Scoping Review checklist (Additional file 1) [32].

### Research questions

What is reported in the literature regarding learning styles, learning style outcome measures and different teaching and learning approaches for PTs? What are the demographics of the included studies and what are the gaps in the literature relating to PT learning styles?

### Search strategy

Nine electronic databases (CINAHL, BIOMED CENTRAL, Cochrane, Web of Science, PROQUEST, PubMed, OTseeker, Scopus, ERIC) were searched from inception to April 2018. One reviewer searched these electronic databases, using key words and MESH terms as appropriate. No limits were set on publication date, study design or country of origin.

### Search terms

The keywords applied to the search included “learning styles” AND (“Allied Health Occupations” OR “physiotherapy” OR “physical therapy”). Table 1 provides an example of the search strategy.

**Table 1:** Search strategy example

	Search strings
#1	“learning styles” AND “Allied Health Occupations”[Mesh] Filters: English
#2	(“physical therapists”[MeSH Terms] OR “physical therapists”[All Fields] OR “physiotherapists”[All Fields]) AND “learning styles” Filters: English

### Inclusion criteria

This review sought English-language, full text primary research articles of any research design. Eligible studies included those assessing the learning styles of undergraduate and post-graduate students, and physiotherapists seeking continuing professional development, all of whom will generally be adults (18 years or older).

### Hierarchy of evidence

Study hierarchy of evidence was determined using the National Health and Medical Research Council (NHMRC) hierarchy of evidence [33].

### Critical appraisal

In line with the scoping review framework, there was no critical appraisal undertaken.

### Data extraction

Data was extracted into a purpose-built MS Excel spreadsheet using the headings: Author (year), country; Article name; Sampling population; Aims; Study design; Underlying learning style theories or models; Outcome measures; and Findings.

### Decision making regarding evidence inclusion

All authors collaborated on the inclusion and exclusion criteria. One reviewer and two health sciences librarians searched the electronic databases. This reviewer identified potentially eligible articles by screening all titles, reading the abstract and determining initial eligibility, finally reading the full text article of potentially eligible studies, and determining final study inclusion. Any concerns on article inclusion were referred to the other reviewers for a decision [31].

### Data analysis

The findings were described narratively in terms of PT learning styles, learning style outcome measures, and possible teaching and learning approaches on learning styles. Gaps in evidence were identified and described, and the available evidence was interrogated to inform ways in which these gaps could best addressed in future research.

## Results

### Article inclusion

**Fig. 2:**
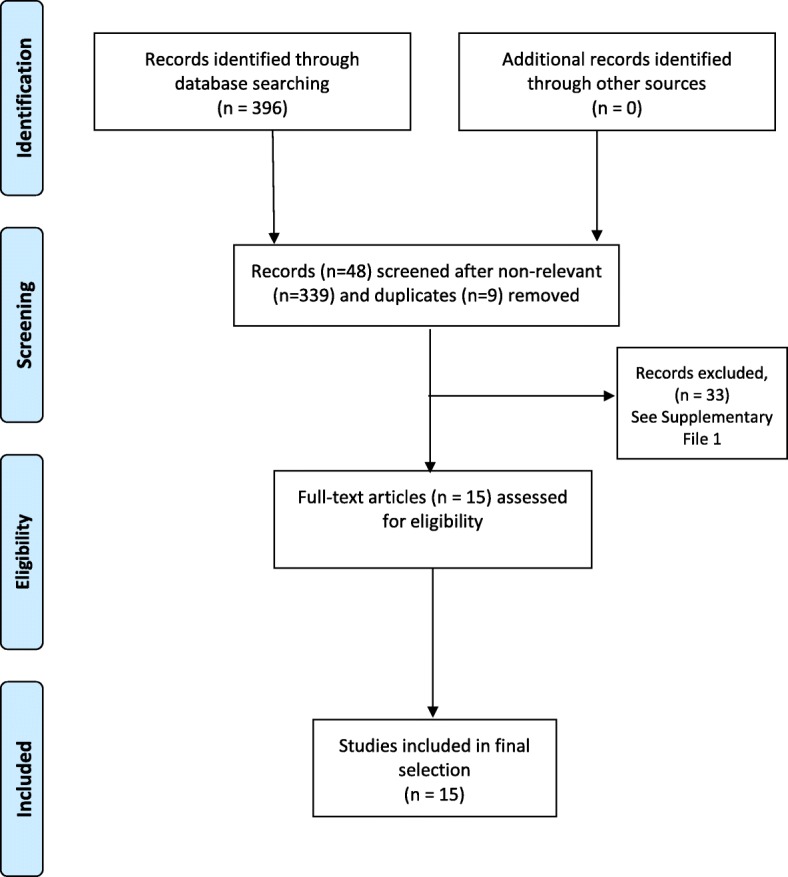
PRISMA flow diagram

396 articles were identified as potentially relevant to the research question. After removing duplicates (*n* = 9) and excluding non-relevant studies (*n* = 339),48 papers were screened for inclusion. Thirty-three studies were excluded with reasons (see Additional file 2) and 15 articles were retained. Figure 2 outlines the PRISMA flow diagram of article selection and inclusion [34]. The included articles reported mostly on physiotherapy undergraduate students (*n* = 910), then post-graduate students (*n* = 361) then physiotherapists seeking continuing professional education (*n* = 23). The physiotherapy participants were either included in the research as a discrete group, or as an identifiable part of a larger cohort of health science disciplines.

### Hierarchy of evidence

All included studies were cross-sectional in design, ranked as III-3 on the NHMRC observational hierarchy of evidence.

### Learning style theories

Table 2 summarises the learning style theories reported in the included studies. Nine studies explicitly stated using the Kolb’s ELT [35–43].

**Table 2:** Summary table of included studies

Author (year)	Country	Sampling population	Learning style theories/ models	Outcome Measures	Learning styles identified
Hauer et al. (2005)	USA	17 PG	Kolb’s ELT	LSI-IIa	Converger
Milanese et al. (2013)	Australia	48 UG	Kolb’s ELT	LSI (V3.1)	Converger/ Assimilator/ Accommodator
Zoghi et al. (2010)	Australia	49 UG	Kolb’s ELT	LSI	Converger
Brown et al. (2008)	Australia	60 UG	None noted	LSI (V3); VARK	Assimilator; Kinesthethic (VARK)
Al Maghraby & Alshami (2013)	Saudi Arabia	53 UG	Kolb’s ELT	LSQ	Concrete-sequential PLS; “Hands-on” TM
Rai & Kahtri (2014)	India	12 UG	None noted	VARK	Kinaesthetic (VARK)
Hess & Frantz (2014)	South Africa	177 UG	None noted	ILS; LSQ	Visual-verbal (ILS); Kinaesthethic (LSQ)
Katz & Heimann (1991)	Israel	57 UG; 23 PT practitioners	Kolb’s ELT	LSI	Converger; Assimilator
Olson & Scanlan (2002)	USA	190 PG	Gregorc model of cognition	Gregorc style delineator	Dual learning style; concrete-sequential
Mountford et al. (2006)	Australia	164 UG; 42 PG	Honey and Mumford model	Honey & Mumford’s LSQ	Reflector
Manee et al. (2013)	Bahrain	82 UG	Kolb’s ELT	LSI-IIa	Assimilator (AC)
Wessel et al. (1999)	Canada	158 UG	Kolb’s ELT	LSI	Converger
Wessel et al. (2004)	Canada	94 PG	Kolb’s ELT	LSI	Converger; Assimilator
Brudvig et al. (2015)	USA	18 PG	Kolb’s ELT	LSI4.0	Experiencing, reflecting, analysing, thinking, balancing
Smith et al. (2006)	USA	50 UG	Gregorc model of cognition	Gregorc style delineator	Dual learning style; concrete-sequential

One study applied Honey and Mumford’s approach [44]; two studies used the Gregorc model of cognition [22, 45], and three studies did not report any underlying theory [46–48].

### Learning styles

There was consistency in the included literature regarding PT learning styles. The most preferred learning styles of PT learners, according to Kolb’s ELT, were Converger [37, 38, 41–43] or Assimilator [36, 39, 41, 43, 46]. PT learner’s least preferred learning styles were Diverger [40, 42] and Accommodator [42, 46].

Katz and Heimann (1991) reported that PT professionals undertaking professional development were more prone to using an Assimilator style [38], whilst studies utilising the VARK (visual-aural-read/write-kinesthetic) or Learning style questionnaire (LSQ) [35, 46–48] found the preferred PT learning style to be kinaesthetic learning. Studies utilising the Gregorc model of cognition [22, 45], found the preferred learning style was a dual learning style, closely followed by a concrete sequential learning style.

### Learning style outcome measures

Table 2 also outlines the reported learning style outcome measures. These included different versions of the LSI [36–43, 46]; VARK questionnaire [46, 48]; Gregorc style delineator [22, 45]; Honey and Mumford’s LSQ [44, 47]; and the Index of Learning Styles (ILS) [47].

### Demographics of studies

Table 2 also summarises the country of origin and sample population of the included studies. The included studies came mostly from developed countries including Australia [40, 42, 44, 46], USA [22, 36, 37, 45], Canada [41, 43]; and one each from Saudi Arabia [35], India [48], South Africa [47], Israel [38] and Bahrain [39]. Two studies were conducted between 1991 and 1999 [38, 41]; six studies were conducted between 2002 and 2008 [22, 37, 43, 45, 46]; and seven studies were conducted between 2010 and 2015 [35, 36, 39, 40, 42, 47, 48]. Nine studies reflected undergraduate learners [22, 35, 38, 39, 41, 42, 44, 46–48]. Four studies reported on post-graduate students [36, 37, 43, 45], and one [38] reported on the PT professionals.

## Discussion

This review aimed to identify and describe the learning styles of PT adult learners. This review highlighted some consistency in the literature regarding PTs preferred learning styles, and in the way that learning styles were measured, teaching conceptualised or delivered, or outcomes assessed. No experimental studies were identified, thus examination of the effectiveness of different learning approaches could not be determined.

This review also highlighted that there is no “one-size-fits-all” approach to the education of PT learners, particularly professional PT learners wanting to further their own professional development. This review identified a modest evidence base, comprising 15 observational studies from eight countries, published in the last 26 years, with heterogeneous samples of PT learners. PT learners largely reflected undergraduate students (*n* = 910). The much smaller samples of postgraduate students, and PT professionals preclude clear understanding of how learning styles influence the way PT learners learn. When reflecting on the fact that the studies focus mainly on undergraduate students, one has to consider how undergraduate students have learnt over the 26 year time span of the review. These students would have spanned the pre-social media phase up to the current undergraduate population, where many of them will source their information electronically. In a recent systematic review, it was found that, even though it seems that generation Y learn differently, the evidence is still inconsistent as to how they learn differently and if it was much different to the previous generation X [49].

The gaps identified in this scoping review indicated that any new training program for PT learners (particularly professionals seeking professional development) needs to be conceived on a modest observational study evidence-base, which provides less than adequate guidance for the best ways to construct curriculum, and assess learners’ needs, and outcomes from training.

### Learning styles

The lack of consistency in how PT learning styles were described and assessed was evidenced using three different approaches and the lack of comparability between the different approaches. For instance, the two most common learning style theories identified in the review (Kolb and Gregorc) do not share similar domains, and therefore it is difficult to correlate the findings between studies. In the nine studies that applied Kolb’s approach, there was consistency about PTs’ learning styles (Convergers and Assimilators). Both learning styles use abstract conceptualisation (thinking) as the dominant learning approach. The Assimilator learning style of PT professionals is similar to other ‘scientific’ professionals, including engineers, physicians and scientists [38, 41]. This is not surprising, as scientific professions rely on abstract conceptualisation through meaning creation, planning from past experiences and reflective observation on previous experience [50]. This assists in synthesising information sources and applying this to solve practical problems. This links with Convergers that is based on preference for interacting with, and solving of, problems [50]. This may also support PTs’ approach to diagnosing and treating patients, if they perceive the patient’s condition as a “problem” that can be solved.

Olson and Scanlan (2002) and Smith et al. (2006) used Gregorc’s model of cognition as underpinning theory. Both found “dual” learning style and concrete sequential approaches (interacting with defined reality in a step-by-step manner) [22, 45]. This seems comparable to the Convergers and Assimilators, as the learning takes place logically, through interacting with problems systematically. This observation is only based on the similarity in description between the two approaches and not based on direct comparison between domains.

Assessment of PTs’ learning styles in the studies utilising VARK or LSQ found a preferred learning style of kinaesthetic learning [35, 46–48]. Although no direct comparisons could be made between the two learning style approaches, it appears that kinaesthetic learning is similar to Kolb’s Converger, again due to its description in the literature. In this approach, PT learners take a “hands-on” manner to learn optimally. In previous studies, students that had a kinaesthetic learning style seemed to perform better academically, due to them employing deep learning approaches, a concept that entails students engaging with higher learning material to better solve a problem or complete a task [51, 52].

### Learning style outcome measures

Researchers seeking guidance regarding the best outcome measure to choose, would not find it from this review. Nine studies reported using the Kolb’s ELT, also used the Kolb LSI, albeit different versions of it. This precluded synthesis of findings from this subset of studies. The LSI-version 1 (created in 1969) showed low internal and test-retest reliability. The progression from LSI2 (published in 1985), LSI2a (published in 1993) to LSI3 (published in 1999) and LSI3.1 (published in 2005), lead to improved internal and test-retest reliability [17]. The KLSI4.0 was expanded into a nine-learning style typology and assesses learning flexibility and reports on how to improve learning. It also states improved psychometric properties [53]. However, Manolis et al. (2013) questioned the usefulness of a single identified learning style as individuals may have different preferred learning styles in different circumstances and the psychometric properties of the KLSI, including low reliability and predictive powers [16].

Honey and Mumford’s LSQ, reported by Mountford et al. (2006), was found to have modest internal consistency reliability, and seems more appropriate in business management training than higher education, particularly undergraduate business and health sciences degrees [54]. Thus, it appears that there is opportunity to further investigate this outcome measure for PT learners.

The VARK questionnaire, reported in two studies [46, 48], has been shown to have adequate validity, however there may be possible wording and scoring problems which could impact on the usefulness of this measure in research [55]. The researchers found that item wording in some instances were directed towards participants with higher economic means, making said items of little use in populations with lower economic circumstances. The scoring problems partly arises from the VARK algorithm, allowing for multiple learning style preferences and not clearly stating how the learning style preferences are classified as “very strong, strong or mild” [55].

Gregorc’s style delineator, reported by Smith et al. (2006) and Olson and Scanlan (2002), has been found to have low internal consistency and the combined values of the instrument may lead to the incorrect representation of the preferred learning style [56]. The Felder Silverman ILS was reported by Hess and Franz (2014). The instrument was found to have acceptable internal reliability and construct validity [57]. However, the evidence base for this instrument is scant, and further research is required to further test this outcome measure.

### Teaching and learning approaches

One study [22] investigated the relationship between learning styles and delivery of teaching material. It found that learning styles did not influence student performance of either group, one receiving multimedia instruction and one receiving live instruction in undergraduate PT learners. The multimedia group did however, score higher on written and practical examinations, due to students being able to tailor learning materials to their preferred learning method. The undergraduate PT learners’ preferred teaching method of “hands-on training” with well-structured, organised programs for instructional activities is consistent with kinaesthetic and Converger learning styles [35, 45]. Wessel et al. (1999) found no relationship between problem-solving ability and learning style [41]. Moreover, Wessel et al. (2004) found no difference in critical thinking abilities among different preferred learning styles in post-graduate learners [43]. However, this contrasts with Brudvig et al. (2015) who reported that most post-graduate learners favouring abstract conceptualisation learning style, scored higher on the Health Science Reasoning Test. There is thus no consensus whether learning style preference, critical thinking or problem-solving skills may be linked.

### Demographics of studies

‘Culture’ potentially plays a significant role in how people learn, and it can be considered in terms of national and professional characteristics [24]. There were few differences in findings between countries. Considering the different ways that learning styles were measured in the included studies, it appears that the same teaching and learning approach would be acceptable to students in any country, as long as it includes active learning, problem solving and hands-on activities. Considering professional characteristics, similarities in learning styles across countries may have to do with institutional collectivism, that is “the degree to which organizational and societal institutional practices encourage and reward collective distribution of resources and collective action” (p.12) [58]. This might explain similarities in learning styles identified in this review, despite research being conducted using different theoretical frameworks, on diverse groups of PT learners, in quite different countries. The consistencies potentially reflect the nature of the PT profession (active, doing whilst reflecting [15]) and characteristics of the type of person who studies and practises physiotherapy.

### Further research opportunities

This review highlighted the need for further research into:

- defining and describing PT learning styles in a way to be used as an industry standard;
- developing valid and reliable learning style outcome measures applicable across PT learners and settings;
- assessing the impact of learning styles on learning outcomes in varying teaching styles
- developing curricula based on current best evidence of how PT learners learn; and
- constructing effective training programs for adult PT learners that map to best practice outcome measures.

### Limitations

Whilst scoping reviews are inherently limited in what they can produce (i.e. no critical appraisal, limited independent investigator engagement), this review highlighted the importance of taking a ‘scoping’ approach to understand the directions that the literature in PT learning styles have taken over the last 26 years. The modest observational study evidence-base with its heterogeneous study populations, provides limited guidance for the optimum construction of curricula to address learners’ needs and preferences for learning opportunities, particularly PT professionals seeking professional development. With an increased awareness of continuing professional development, but with limited evidence on the PT professionals’ preferred learning styles, teaching material may be ineffective to assist in professional development.

## Conclusion

This review identified the need for further research into PT adult learning styles, valid and reliable learning style outcome measures, and teaching and learning approaches to affect different learning styles. Despite the heterogeneity of the included literature, it seems reasonable to conclude that PTs seem to have similar preferred learning styles, these being active participation in learning activities that are underpinned by clear theoretical concepts. This information provides some guidance for constructing potentially-effective training programs for PT learners, whereby learning activities need to be aligned to ensure the learners are given the necessary theoretical backing and then giving them enough opportunity to practice and apply the theory. These programs should minimise lecture-type activities and optimise time spent on problem-solving and practical knowledge application, possibly through blended learning activities. Whatever the choice of learning outcome measures for research into the effectiveness of PT training programs, it seems sensible to match the learning style framework underpinning the training material, and capture it in the most sensitive and reliable manner, ways in which participating in training have impacted on PT learners’ experiences. More research is needed in developing countries, and on postgraduate and professional physiotherapy learners.

## Additional files

Additional file 1: PRISMA-ScR checklist. This supplementary file supplies the reporting process that was followed according to the PRISMA Extension for Scoping reviews checklist. (DOC 62 kb)

Additional file 2: Articles excluded after applying inclusion and exclusion criteria. This additional file supplies the reasons for exclusion of each article after applying inclusion and exclusion criteria. (DOCX 15 kb)

## Abbreviations

AC: Abstract conceptualisation
AE: Active experimentation
AH: Allied Health
CE: Concrete experience
ELT: Experiential learning theory
ILS: Index of learning survey
KLSI: Kolb’s Learning style inventory
LSI: Learning style inventory
LSQ: Learning style questionnaire
PT: Physiotherapy (ist)
RO: Reflective observation
USA: United States of America
VARK: visual-aural-read/write
