# Supplementary material for: Learning styles of physiotherapists: a systematic scoping review
Source: BMC Med Educ. 2019 Jan 3;19:2. doi: 10.1186/s12909-018-1434-5 (PMC6318981; doi:10.1186/s12909-018-1434-5)
Supplement: Supplementary file 2 — Articles excluded after applying inclusion and exclusion criteria. This additional file supplies the reasons for exclusion of each article after applying inclusion and exclusion criteria. (DOCX 15 kb) [file 12909_2018_1434_MOESM2_ESM.docx]

**Supplementary File 1: Excluded articles with reasons**

| **Author** | **Year** | **Reason** |
| --- | --- | --- |
| Rogers & Hill | 1980 | Focus on OTs, no PTs included |
| Vittertoe & Hooker | 1983 | Focus on nursing students, no PTs |
| Vittertoe | 1983 | Article could not be retrieved |
| Cross & Tilson | 1997 | Identifying different tools for learning style assessment, not in scope of review |
| Farina | 1997 | Doctoral thesis on learning characteristics not in scope of review |
| Tsang et al | 1997 | Article could not be retrieved |
| Linares | 1999 | No subdivision of physiotherapists |
| Daniel | 1999 | Doctoral thesis on learning styles not in scope of review |
| Sandmire et al | 2000 | Article could not be retrieved |
| Sellheim | 2001 | Beliefs of PT faculty not in scope of review |
| Rennie | 2002 | Doctoral thesis on learning styles not in scope of review |
| Hauer | 2002 | Doctoral thesis on learning styles not in scope of review |
| Sandmire & Boyce | 2004 | No subdivision of physiotherapists |
| Mayya et al | 2004 | Learning approaches not in scpoe of review |
| Goulet & Owen-Smith | 2005 | Types of learning not in scope of review |
| DiBartola | 2006 | No subdivision of physiotherapists |
| Thomas & Gaden | 2006 | Conference presentation not in scope of review |
| Sellheim | 2006 | Beliefs of PT faculty not in scope of review |
| Gleeson | 2007 | PT professionalism not in scope of review |
| Secomb | 2008 | Teaching methods not in scope of review |
| Healey | 2008 | PT learning approach not in scope of review |
| Gunn & Goding | 2009 | Continuous professional development of PTs not in scope of review |
| Greiner | 2011 | Doctoral thesis on learner satisfaction not in scope of review |
| Castro-Sánchez et al | 2012 | Focus on problem-based learning, not learning styles |
| Gummesson & Nordmark | 2012 | Reflective practice not in scope of review |
| Maloney et al | 2013 | Learner attitudes not in scope of review |
| Good et al | 2013 | No subdivision of physiotherapists |
| Sjödahl Hammarlund et al | 2013 | PT experience of Inquiry-based learning not in scope of review |
| Heath | 2013 | Book on learning theories of PT not in scope of review |
| Paterson & Chapman | 2013 | Reflective practice not in scope of review |
| Weggelaar-Jansen et al | 2015 | No subdivision of physiotherapists |
| Greenfield | 2015 | Narrative as education method not in scope of review |
| Farkas et al | 2016 | Focus on anatomy and physiology students, no PTs |
| Key: OT - occupational therapy; PT - Physiotherapy; PTs - physiotherapists | | |
